# Overexpression of SSB_Xoc_, a Single-Stranded DNA-Binding Protein From *Xanthomonas oryzae* pv. *oryzicola*, Enhances Plant Growth and Disease and Salt Stress Tolerance in Transgenic *Nicotiana benthamiana*

**DOI:** 10.3389/fpls.2018.00953

**Published:** 2018-07-05

**Authors:** Yanyan Cao, Mingtao Yang, Wenxiu Ma, Yujing Sun, Gongyou Chen

**Affiliations:** ^1^School of Agriculture and Biology, State Key Laboratory of Microbial Metabolism, Shanghai Jiao Tong University, Shanghai, China; ^2^State Key Laboratory of Crop Biology, College of Life Sciences, Shandong Agricultural University, Tai’an, China

**Keywords:** transgenic *N. benthamiana*, *Ssb_Xoc_*, plant growth, hypersensitive response, pathogen resistance, stress tolerance

## Abstract

We previously reported that SSB_Xoc_, a highly conserved single-stranded DNA-binding protein from *Xanthomonas* spp., was secreted through the type III secretion system (T3SS) and functioned as a harpin-like protein to elicit the hypersensitive response (HR) in the non-host plant, tobacco. In this study, we cloned *Ssb_Xoc_* gene from *X. oryzae* pv. *oryzicola* (*Xoc*), the causal agent of bacterial leaf streak in rice, and transferred it into *Nicotiana benthamiana* via Agrobacterium-mediated transformation. The expression of *Ssb_Xoc_* in transgenic *N*. *benthamiana* enhanced growth of both seedling and adult plants. When inoculated with the harpin Hpa1 or the pathogen *Pseudomonas syringae* pv. *tomato* DC3000 (*Pst* DC3000), the accumulation of reactive oxygen species (ROS) was increased more in *Ssb_Xoc_* transgenic lines than that in wild-type (WT) plants. The expression of pathogenesis-related protein genes (*PR1a* and *SGT1*), HR marker genes (*HIN1* and *HSR203J*) and the mitogen-activated protein kinase pathway gene, *MPK3*, was significantly higher in transgenic lines than in WT after inoculation with *Pst* DC3000. In addition, *Ssb_Xoc_* transgenic lines showed the enhanced resistance to the pathogenic bacteria *P*. *s*. *tabaci* and the improved tolerance to salt stress, accompanied by the elevated transcription levels of the defense- and stress-related genes. Taken together, these results indicate that overexpression of the *Ssb_Xoc_* gene in *N*. *benthamiana* significantly enhanced plant growth and increased tolerance to disease and salt stress via modulating the expression of the related genes, thus providing an alternative approach for development of plants with improved tolerance against biotic and abiotic stresses.

## Introduction

Plants are exposed to diverse stress conditions throughout their life cycle, including biotic and abiotic stresses. To cope with biotic stress, plants employ innate immune systems to overcome the microbial invasion (Jones and Dangl, 2006; Thomma et al., 2011). The first line of defense is induced by pathogen-associated molecular patterns (PAMPs), which includes a diverse group of molecules such as flagellin (Felix et al., 1999), EF-Tu (Kunze et al., 2004), chitin and harpins (He et al., 1993; Zou et al., 2006). Harpins are glycine-rich, heat-stable and protease-sensitive proteins that are secreted through the type III secretion system (T3SS) (Wei et al., 1992). Previous researches have demonstrated that plants are highly sensitive to harpin elicitors. The harpins stimulate the hypersensitive cell death, the oxidative burst and the expression of defense-related genes (He et al., 1993; Andi et al., 2001; Ichinose et al., 2001), and activate the mitogen-activated protein kinase (MAPK)-dependent signaling pathway (Desikan et al., 1999, 2001; Lee et al., 2001), which finally induce the defence response in plants.

Previous studies have shown that treatment with harpins induces plant growth (e.g., stimulates the elongation of roots) and enhances resistance to aphids in *Arabidopsis* (Dong et al., 2004; Lü et al., 2011, 2013). Up to now, multiple harpins have been expressed in plants, including *Arabidopsis*, rice, wheat, tobacco, cotton, and soybean, and the transgenic plants exhibited enhanced plant growth and improved resistance to pathogens (Jang et al., 2006; Shao et al., 2008; Miao et al., 2010; Choi et al., 2012; Wang D. et al., 2014; Du et al., 2018). For example, the transformation of cotton with *hpa1* enhanced the defense response against *Verticillium dahliae* (Miao and Wang, 2013; Zhang et al., 2016). Furthermore, the heterologous expression of a functional fragment of the harpin protein Hpa1_Xoo_ induced phloem-based defense against the English grain aphid in wheat (Fu et al., 2014). In addition, the expression of harpins also improves tolerance to abiotic stress. Previous studies demonstrate that HrpN increased drought tolerance by activating abscisic acid (ABA) signaling in *Arabidopsis*, and the harpin-encoding gene, *hrf1*, increased tolerance to drought stress in rice (Dong et al., 2005; Zhang et al., 2011). Recent studies indicate that overexpression of the harpin-encoding gene, *popW*, enhances plant growth and resistance to *R*. *solanacearum*, and also increases drought tolerance in transgenic tobacco (Wang C. et al., 2014; Wang et al., 2016; Liu et al., 2016). Increasing evidence shows that the multiple effects of harpins can be attributed to cross-talk of distinct signaling pathways to regulate development and defense in plants (Chen et al., 2008).

SSBs are highly conserved single-stranded DNA-binding proteins that protect ssDNA from nucleolytic digestion (Fedorov et al., 2006). We recently demonstrated that the SSB protein from *Xanthomonas oryzae* pv. *oryzicola* (*Xoc*) was shown to function as a harpin in tobacco (e.g., elicited an HR). Furthermore, treatment with SSB_Xoc_ improved plant growth and resistance to the fungal pathogen *Alternaria alternate* in *Nicotiana tabacum* cv. Xanthi (Li et al., 2013). In this study, the gene encoding *Ssb* in *X*. *oryzae* pv. *oryzicola* was transformed into *N. benthamiana*. Our research displays that *Ssb_Xoc_* transgenic plants exhibit enhanced plant growth, improved pathogen resistance, and increased tolerance to salt stress. To our knowledge, up to now there are no prior reports showing that the overexpression of harpins can enhance salt tolerance.

## Materials and Methods

### Generation of *Ssb_Xoc_* Transgenic *N. benthamiana* Plants

**FIGURE 1:**
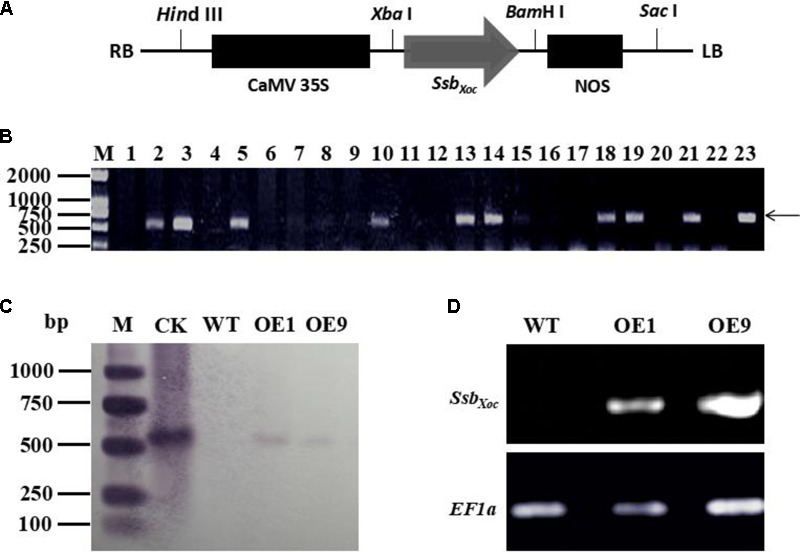
Identification of *Ssb_Xoc_* transgenic *N. benthamiana* plants. **(A)** Construction of vector expressing *Ssb_Xoc_* in transgenic *N. benthamiana*. The CaMV promoter and nopaline synthase (NOS) polyadenylation signal are shown in black solid rectangles and flanked the *Ssb_Xoc_* coding region. **(B)** PCR analysis of transgenic lines using the *Ssb_Xoc_-*specific primers. Lane M, molecular weight marker; lanes 1-22 represent different transgenic lines, and lanes 2 and 21 are lines OE1 and OE9, respectively. Lane 23 contains the positive control, and the arrow shows the location of the 552-bp *Ssb_Xoc_* PCR product. **(C)** Southern blot hybridization of transgenic lines, OE1 and OE9, with dig-labeled *Ssb_Xoc_*. Lanes: M, molecular weight marker; CK, check, pCAMBRIA2300-*Ssb_Xoc_*; WT, wild-type *N. benthamiana*; OE1 and OE9, transgenic lines containing *Ssb_Xoc_* gene. **(D)** Expression measurement of *Ssb_Xoc_* gene in transgenic lines OE1 and OE9 by RT-PCR. The housekeeping gene, *EF1α*, was used as an internal control for normalizing the data.

Full-length *Ssb_Xoc_* gene (552 bp) was amplified by PCR using the specific primers (**Table 1**). The amplified product was cloned into pMD18-T Simple Vector (TaKaRa, Dalian, China) and then subcloned into the binary vector pCAMBIA2300 at *Xba*I and *Bam*HI sites, which were placed downstream of the constitutive cauliflower mosaic virus 35S promoter (CaMV35S) and upstream of the polyadenylation signal of the nopaline synthase terminator (NOS) (**Figure 1A**). The recombinant clone, pCAMBIA2300-*Ssb_Xoc_*, was then transferred into *Agrobacterium tumefaciens* EHA105 for transformation of *N. benthamiana.* The *Ssb_Xoc_* transgenic plants were determined by PCR amplification with the specific primers of *Ssb_Xoc_* till T_2_ generation.

**Table 1:** Primers designed and used for PCR.

Genes	Primer sequences (5′ – 3′)		Purpose
	Forward	Reverse	
*Ssb_Xoc_*	CGGGATCCATGGCCCGCGGCATCAATAAAGT	CCTCTAGATCAGAACGGGATATCGTCGTCGGC	Cloning
*Ssb_Xoc_*	ATGGCCCGCGGCATCAATAAAGT	TCAGAACGGGATATCGTCGTCGGC	RT-PCR, Probe
*EF1α*	AGACCACCAAGTACTACTGCAC	CCACCAATCTTGTACACATCC	RT-PCR
*Ssb_Xoc_*	CAGGGTGATGGTGGATACGG	ATATCGTCGTCGGCGAAATC	qRT-PCR
*PR1a*	GGTGTAGAACCTTTGACCTGGG	AAATCGCCACTTCCCTCAGC	qRT-PCR
*PR2*	TAGAGAATACCTACCCGCCC	GAGTGGAAGGTTATGTCGTGC	qRT-PCR
*PR4*	GTGACGAACACAAGAACAGGAA	CCACTCCATTTGTGTCCAAT	qRT-PCR
*SGT1*	CCTTCTATGAGCAGACATCCCA	GCGTCCAGTATGACAACCCA	qRT-PCR
*HIN1*	TGCGTCCAGTATTCAAAGGTCA	GCTTCACTTCCATCTCATAAACCC	qRT-PCR
*HSR203J*	TGCGTCCAGTATTCAAAGGTCA	GCTTCACTTCCATCTCATAAACCC	qRT-PCR
*MPK3*	CGGCACATGGAACACG	GACCGAATAATCTGATGAAGG	qRT-PCR
*APX*	TGGAACCCATCAAGGAGCAG	ATCAGGTCCTCCAGTGACTTC	qRT-PCR
*GPX*	GTTTCCGCTAAGAGATTTGAGTTG	CCCTTAGCATCCTTGACAGTG	qRT-PCR
*CAT1*	AACAAGGCTGGGAAATCAACC	TGGCTGTGATTTGCTCCTCC	qRT-PCR
*EXPA1*	TTGTTTCTCTGCTTCTGGATGG	CTTAATGCAGCAGTGTTTGTACCA	qRT-PCR
*EIN2*	GGCATAATAGATCTGGCATTTTCC	TATCTAAGAGCATCGGTGCAGTTG	qRT-PCR
*EF1α*	AGACCACCAAGTACTACTGCAC	CCACCAATCTTGTACACATCC	qRT-PCR

### Plants and Growth Conditions

Seeds of WT and *Ssb_Xoc_* transgenic lines OE-1 and OE-9 (T2 generations) were surface-sterilized with 75% ethanol and 10% sodium hypochlorite for 0.5 and 5 min, respectively. They were then separately transferred to Murashige and Skoog (MS) medium without or with 100 mg L^-1^ kanamycin and cultivated in a light-controlled incubator at 25°C. Fifteen days later, the seedlings were transplanted to pots and grown in a greenhouse with a 16-h light/8-h dark photoperiod with 50% relative humidity at 25°C.

### Plant Growth Analysis

The root lengths of transgenic lines (T2 generations) and WT plants grown in MS medium were measured after 15 days. Three independent experiments were performed and at least 20 seedlings were analyzed in each experiment. The phenotypes of plants were determined after the seedlings were transplanted to pots and cultivated for 4 weeks.

### Bacterial Strains and Growth Conditions

Bacterial strains used in this study were *Pst* DC3000 and *P*. *s*. *tabaci*. Both of them were grown at 28°C on King’s medium B (KMB) with or without rifampicin, respectively. They were resuspended and diluted to the appropriate concentration with 10 mM MgCl_2_ for subsequent research.

### Determination of ROS Levels

Fully developed leaves of 2-month-old WT and T2 *Ssb_Xoc_* transgenic plants were separately injected with 100 μl Hpa1 protein (10 μg ml^-1^)and *Pst* DC3000 (OD_600_ = 0.01) using 1-mL needleless syringes. After 6 h, treated leaves were collected and incubated in diaminobenzidine (DAB) for 8 h at 25°C and then were immersed in boiling ethanol (95%) for 10 min to remove the dye (Thomas and Lemmer, 2005). After further incubation in 60% ethanol for 4 h, photographs were taken for visualization of reactive oxygen species (ROS). To quantify ROS accumulation, treated samples were collected separately at 0 and 6 hpi for detection of H_2_O_2_ contents as described previously (Bernt and Bergmeyer, 1974; Cao et al., 2015).

### Bacterial Growth Analysis

The fully expanded leaves of 2-month-old WT and T2 *Ssb_Xoc_* transgenic lines were inoculated with *P*. *s*. *tabaci* (OD = 0.01), and the phenotypes were photographed at 36 hpi. In order to quantify the bacterial growth, the plants were inoculated with 10^5^ CFU/ml *P*. *s*. *tabaci* as described previously (Klement, 1963; Thilmony et al., 1995). Briefly, a *P. s. tabaci* strain was grown overnight in KMB, washed twice, and resuspended at the appropriate concentration in 10 mM MgCl_2_. And bacterial suspensions were then infiltrated into fully developed leaves using 1-mL needleless syringes. To determine bacterial growth in plants, 1 cm^2^ leaf disks were excised from the inoculated tissue of each treatment at 0, 1, and 2 dpi. The bacterial populations in the leaves were determined by plating serial dilutions on KMB.

### RNA Isolation and Gene Expression Analysis

Total RNA was isolated from leaves of WT and *Ssb_Xoc_* transgenic plants (T1 and T2 generation) using TRIzol reagent (TaKaRa, Japan) as recommended by the manufacturer. RT-PCR with gene-specific primer pairs was performed to evaluate the expression of *Ssb_Xoc_* in WT and transgenic plants. The expression of *Ssb_Xoc_* and genes related to the defense response, oxidative stress, and salt stress was measured using quantitative real-time PCR (qRT-PCR), and all of the primers used in these experiences were listed in **Table 1**. *EF1α* was used as an internal standard in these experiments.

### Southern Blot Analysis

Genomic DNA was extracted from WT and T1 *Ssb_Xoc_* transgenic lines using CTAB as described previously (Murray and Thompson, 1980). The recombinant plasmid pMD18-*Ssb_Xoc_* and genomic DNA were digested with *Bam*HI and *Xba*I enzymes, and fragments were separated by electrophoresis in a 1.3% agarose gel at 80 V for 12 h. DNA was transferred to nylon membranes and hybridized with the *Ssb_Xoc_* PCR product, which was labeled with digoxigenin as recommended by the manufacturer (Dig-Labeling Kit, Roche). Conditions for hybridization and detection were followed as described by Aviv et al. (2011). The primers used for amplifying the *Ssb_Xoc_* probe were listed in **Table 1**.

### Salt Stress Tolerance Assays

To examine germination rates during salt stress, seeds of T2 *Ssb_Xoc_* transgenic lines and WT plants were surface-sterilized and sown on MS medium supplemented with 100 mM NaCl cultivated in a light-controlled incubator with a 14-h light/10-h dark photoperiod at 25°C. Germination rates were assayed after 5 days. For analysis of chlorophyll content, leaf disks (1 cm diameter) were excised from fully expanded leaves and floated separately on solutions containing 0, 200, and 400 mM NaCl for 4 d in the incubator. Chlorophyll contents were measured as described by Porra (2002), Kanneganti and Gupta (2008). Leaves were sampled for the measurements of malondialdehyde (MDA) and proline using previously described methods (Bates et al., 1973; Cao et al., 2014) after treatment with salt for 4 days.

### Statistical Analysis

All experiments were repeated three times. Data were presented as the mean ± SD and analyzed using Excel and SPSS. Tukey’s test (*P* < 0.05) was used to determine significant differences.

## Results

### Generation of *Ssb_Xoc_* Transgenic *N*. *benthamiana*

To quickly determine whether *Ssb_Xoc_* gene was present in transformed *N*. *benthamiana*, potential transgenic plants (T_0_ generation) were initially screened by PCR using the *Ssb_Xoc_*-specific primers. Nine lines were obtained that existed a prominent 552-bp fragment in the genomic DNA, which was the predicted size of *Ssb_Xoc_* gene (**Figure 1B**). Two transgenic lines designated OE1 and OE9 were randomly selected for further characterization. Genomic DNA was extracted from OE1 and OE9 and analyzed by Southern blot hybridization. Both lines contained a 0.55-kb hybridizing fragment that corresponded with the predicted size of *Ssb_Xoc_* gene, and this signal was not detected in WT plants (**Figure 1C**). Thus, both PCR and Southern blot analyses indicated that *Ssb_Xoc_* gene had been incorporated into the genome of OE1 and OE9 transgenic plants. To determine whether *Ssb_Xoc_* was expressed in the transgenic lines, the accumulation of *Ssb_Xoc_* mRNA was evaluated by RT-PCR using *EF1α* as an internal standard. A 552-bp product was amplified from the transgenic lines OE1 and OE9, but not from WT (**Figure 1D**), indicating that *Ssb_Xoc_* gene was successfully expressed in transgenic lines. In addition, to quantify the expression level of *Ssb_Xoc_* gene in transgenic lines, the qRT-PCR experiment was performed using *Ssb_Xoc_* specific primers (**Table 1**). The result showed that the expression level of *Ssb_Xoc_* in OE9 line was higher than that in OE1 line (Supplementary Figure S1).

### Expression of *Ssb_Xoc_* in Transgenic *N. benthamiana* Enhances Plant Growth

**FIGURE 2:**
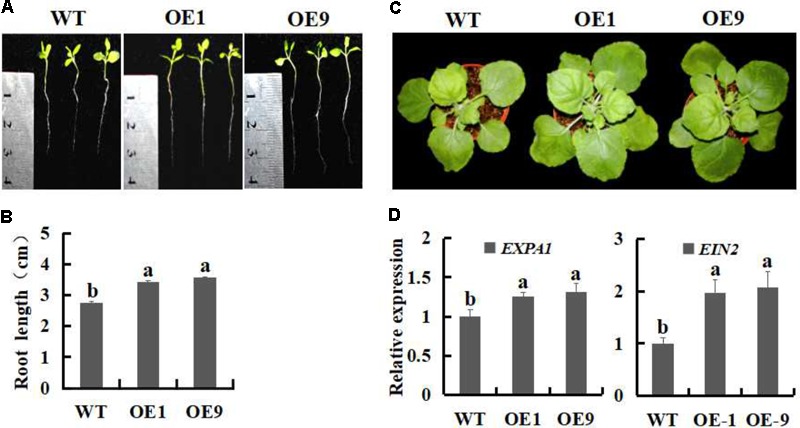
Growth phenotypes of WT and *Ssb_Xo_*_c_ transgenic *N. benthamiana* plants. **(A)** Phenotypes of WT and transgenic lines incubation on MS medium for 15 days. **(B)** Root lengths on MS medium after 15 days. **(C)** Mature-stage phenotypes of plants after 6 weeks. **(D)** Expression analysis of *EXPA1* and *EIN2* by qRT-PCR in mature leaves of WT and transgenic *N. benthamiana*. plants. Error bars represent SD, and values with different letters are significantly different at *P* < 0.05.

To evaluate whether the growth of *Ssb_Xoc_* transgenic plants was enhanced, root lengths were measured after cultivation in MS medium for 15 days. The transgenic lines OE1 and OE9 exhibited increased root lengths as compared with the WT (**Figures 2A,B**), and the difference was significant (*P* < 0.05). Four weeks after transplantation to pots, the transgenic lines still exhibited enhanced plant growth (**Figure 2C**). Previously, Goh et al. (2012) reported that genes in the expansin family, e.g., *AtEXPA1, AtEXPA*5 and *AtEXPA10*, were required for leaf growth, furthermore, the suppression of *AtEXPA* decreased foliar growth in *Arabidopsis*. EIN2 is demonstrate as an essential positive regulator in the ethylene signaling pathway, which is involved in many aspects of the plant life cycle (Johnson and Ecker, 1998; Wang et al., 2002). Thus, we measured the expression levels of expansin-encoding gene, *EXPA1*, and *EIN2*, to investigate whether the transcription of the two genes was enhanced in *Ssb_Xoc_* transgenic plants. As shown in **Figure 2D**, the transgenic lines exhibited higher expression of *EXPA1* and *EIN2* in comparison with WT, which further confirmed the enhanced growth evident in transgenic plants (**Figure 2D**).

### SSB_Xoc_ Improves Defense Responses to Hpa1 and *Pst* DC3000 in Transgenic *N. benthamiana*

**FIGURE 3:**
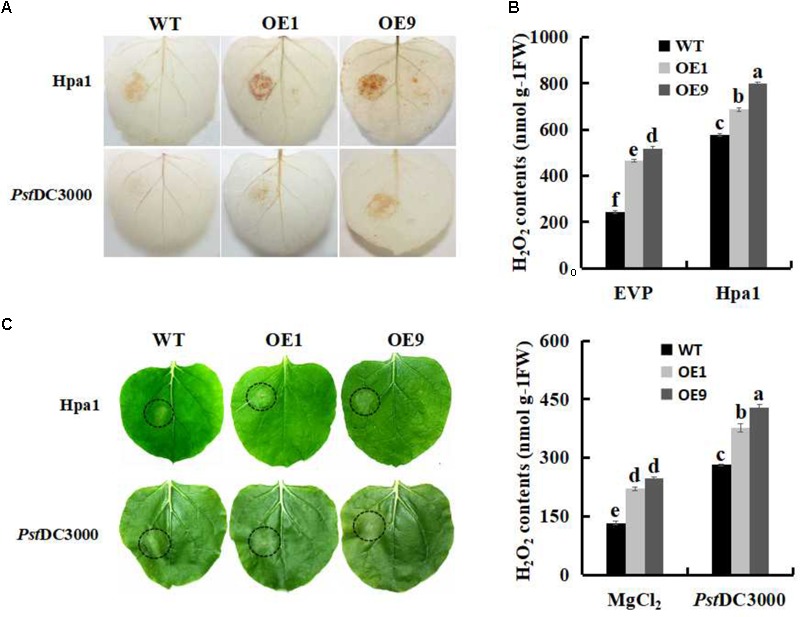
The oxidative burst assay in WT and *Ssb_Xo_*_c_ transgenic *N. benthamiana* plants inoculated with Hpa1 and *Pst* DC3000. WT and transgenic plants were injected with Hpa1 protein (10 μg ml^-1^) and *Pst* DC3000 pathogen (OD = 0.01), and at 6 hpi, treated leaves were collected. **(A)** Visualization of H_2_O_2_ accumulation by DAB staining in leaves inoculated with Hpa1 and *Pst* DC3000. **(B)** Evaluation of H_2_O_2_ levels in leaves. Upper panel shows H_2_O_2_ levels in WT and transgenic lines inoculated with empty vector preparation (EVP; negative control) and Hpa1; Lower panel shows H_2_O_2_ levels in WT and transgenic lines inoculated with 10 mM MgCl_2_ (negative control) and *Pst* DC3000. **(C)** Phenotypes of WT and transgenic lines inoculated with Hpa1 and *Pst* DC3000 after 24 h. Inoculation sites are indicated with open circles. Error bars represent SD, and values with different letters are significant at *P* < 0.05.

The Hpa1 protein and the pathogen of *Pst* DC3000 were individually inoculated to WT and *Ssb_Xoc_* transgenic plants to examine defense responses. DAB staining results indicated that ROS levels were significantly enhanced in *Ssb_Xoc_* transgenic lines as compared with WT (**Figure 3A**). H_2_O_2_ contents were then evaluated to quantify ROS levels in treated leaves. As shown in **Figure 3B**, transgenic lines exhibited higher levels of H_2_O_2_ accumulation than WT plants after inoculation with Hpa1 (**Figure 3B**, upper panel) and *Pst* DC3000 (**Figure 3B**, lower panel).

The accumulation of ROS in response to harpins and incompatible pathogens is generally accompanied by the HR (Zurbriggen et al., 2010). Therefore, WT and *Ssb_Xoc_* transgenic lines were evaluated visually for the HR at 24 hpi. The results showed that, after inoculated with Hpa1 and *Pst* DC3000 for 24 h, WT plants started to appear the HR, while transgenic lines reacted earlier and formed a more prominent HR at the inoculation site (**Figure 3C**), indicating that *Ssb_Xoc_* transgenic plants activated defense response earlier than WT, and this promoted the pathogen resistance.

### SSB_Xoc_ Enhances the Expression of Defense Related-Genes in Transgenic *N. benthamiana*

**FIGURE 4:**
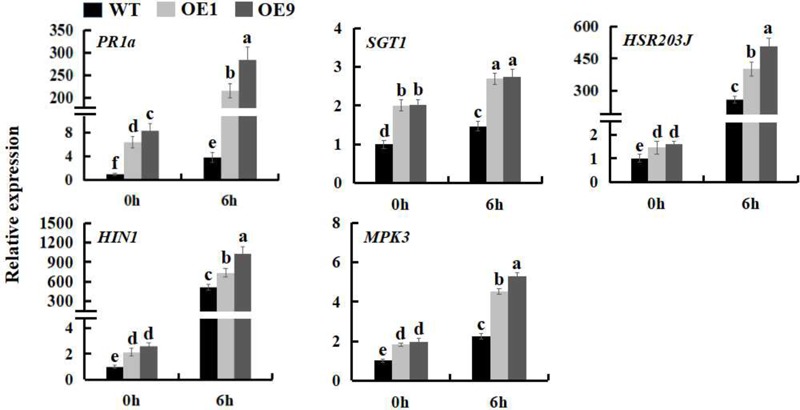
Expression analysis of defense related-genes in WT and *Ssb_Xo_*_c_ transgenic *N. benthamiana* plants inoculated with *Pst* DC3000. Two-month-old seedlings were inoculated with *Pst* DC3000 (OD = 0.01). At 0 and 6 hpi, the leaves were sampled to extract the total RNA to synthesize cDNA, and the transcription levels of *PR1a, SGT1, HIN1, HSR203J* and *MPK3* genes were examined by qRT-PCR. Error bars represent SD, and values with different letters are significantly different at *P* < 0.05.

The expression of many defense genes can be activated during pathogen invasion in plants, including the pathogenesis-related (*PR*) genes, which play an important role in plant defense response (Maurhofer et al., 1994; Van Loon, 1997). To further investigate the mechanism underlying the increased pathogen resistance of *Ssb_Xoc_* transgenic plants, the expression levels of the *PR* genes, *PR1a* and *SGT1*, HR marker genes, *HIN1* and *HSR203J*, and a gene involved in the MAPK-dependent signaling pathway, *MPK3*, were examined during infection by *Pst* DC3000. The results showed that at the time of inoculation with *Pst* DC3000 (0 hpi), the expression of defense-related genes was higher in transgenic lines as compared to WT; at 6 hpi, the expression levels of the five genes were all upregulated in all of the plants, while they were increased more in transgenic lines (**Figure 4** and Supplementary Figure S2), further indicating that *Ssb_Xoc_* transgenic lines could respond more quickly to the invasion of *Pst* DC3000.

### Overexpression of *Ssb_Xoc_* Improves Resistance to *P. s. tabaci*

**FIGURE 5:**
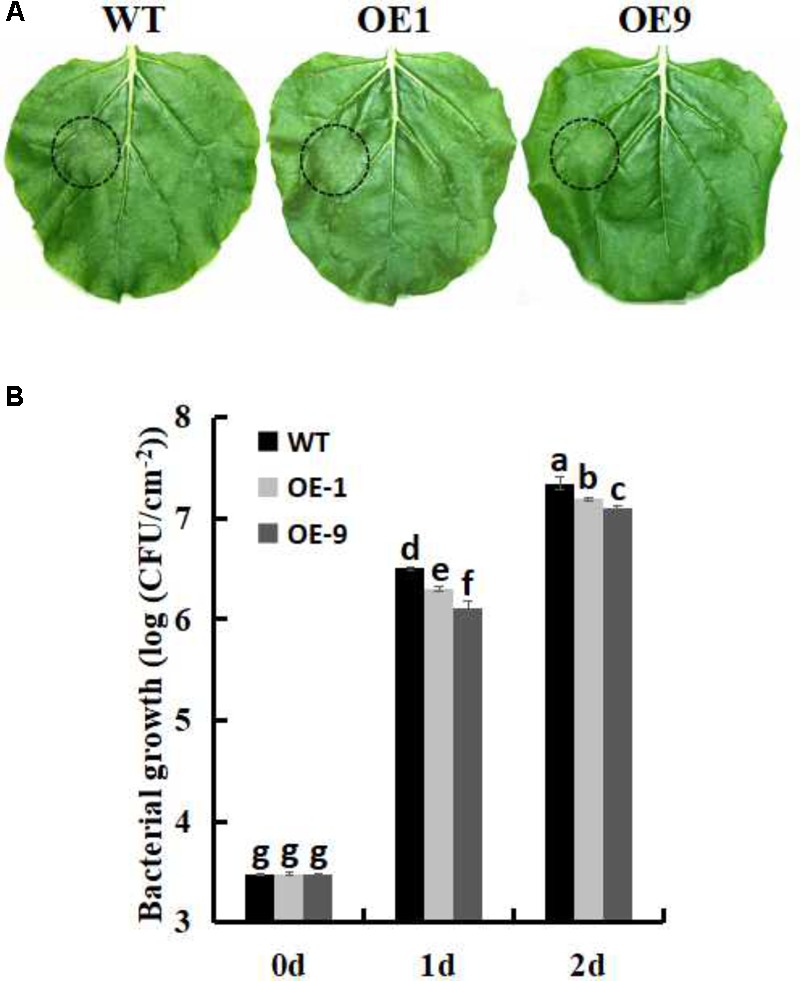
Measurement of bacterial growth in WT and *Ssb_Xoc_* transgenic *N. benthamiana* plants inoculated with *P. s. tabaci*. **(A)** Phenotypic observation of the leaves in WT and transgenic lines after inoculation with *P. s. tabaci* (OD = 0.01) for 36 h **(B)** Bacterial growth in WT and transgenic lines was determined at 0, 1, and 2 days after inoculation with *P. s. tabaci* (10^5^ CFU/ml). Error bars represent SD, and values with different letters are significantly different at *P* < 0.05.

**FIGURE 6:**
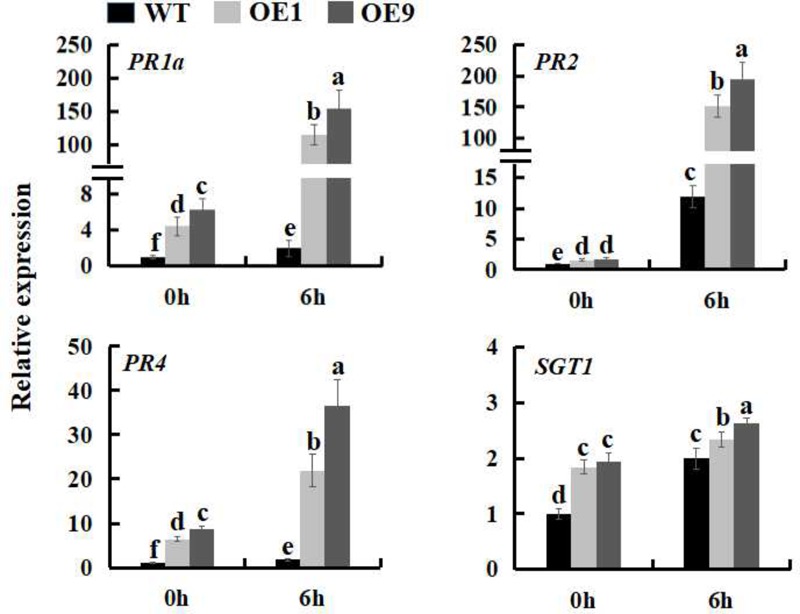
Expression analysis of defense genes in WT and *Ssb_Xo_*_c_ transgenic *N. benthamiana* plants inoculated with *P. s. tabaci*. Two-month-old seedlings were inoculated with *P. s. tabaci* (OD = 0.01). At 0 and 6 hpi, the leaves were sampled to extract the total RNA to synthesize cDNA, and the expression levels of *PR1a, PR2, PR4*, and *SGT1* genes were analyzed by qRT-PCR. Error bars represent SD, and values with different letters are significantly different at *P* < 0.05.

In order to investigate whether *Ssb_Xoc_* transgenic plants could improve bacterial disease resistance, one pathogenic bacteria, *P. s. tabaci*, was used. As shown in **Figure 5A**, *Ssb_Xoc_* transgenic lines displayed less disease symptoms than WT plants at 36 h after inoculation with *P*. *s*. *tabaci* (**Figure 5A**). Correspondingly, the growth of *P. s. tabaci* was significantly lower in transgenic lines than that in WT plants at 1 and 2dpi, respectively (**Figure 5B**), being consistent with the necrosis symptoms in plants. In addition, the expression of defense genes was assayed in WT and *Ssb_Xoc_* transgenic plants after inoculation with *P*. *s*. *tabaci*. The results displayed that *Ssb_Xoc_* transgenic lines showed a higher expression of the pathogenesis-related genes, *PR1a, PR2, PR4* and *SGT1*, than that of WT at the time of inoculation (0 hpi), and at 6 hpi, the expression levels of all the four genes were upregulated, however, they were more higher in transgenic lines than in WT (**Figure 6** and Supplementary Figure S2). All of the above results indicated that *Ssb_Xoc_* transgenic plants had an improvement in resistance to the pathogenic bacterium, *P. s. tabaci*.

### SSB_Xoc_ Enhances Seed Germination and Chlorophyll Retention During Salt Stress

**FIGURE 7:**
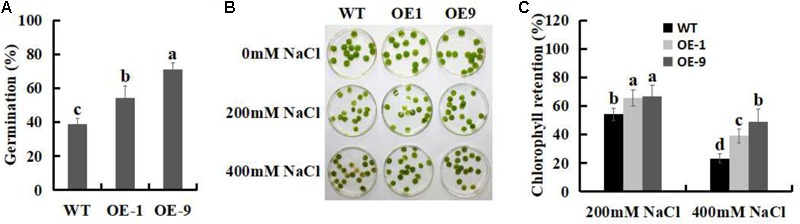
Effects of salt stress on seed germination and chlorophyll content in WT and *Ssb_Xoc_* transgenic *N. benthamiana* plants. **(A)** Analysis of percent seed germination after treatment with 100 mM NaCl for 5 d. **(B)** Phenotypic observation of chlorophyll retention in WT and transgenic leaf disks after treatment with 0, 200, and 400 mM NaCl for 4 d. **(C)** Chlorophyll contents in WT and transgenic plants after treatment with 200 and 400 mM NaCl for 4 d. Error bars represent SD, and values with different letters are significant at *P* < 0.05.

The potential role of SSB_Xoc_ in improving salt stress tolerance was initially investigated by measuring the germination of the seeds after treatment with 100 mM NaCl. As shown in **Figure 7A**, the percentages of seed germination of the two *Ssb_Xoc_* transgenic lines were 54.5 and 71%, respectively, which were significantly higher than WT (38.8%). This result showed that the improved germination rate was most pronounced in OE9 transgenic line (**Figure 7A**).

Chlorophyll retention is used as a physiological indicator of salt tolerance in plants (Sui et al., 2017). In the present study, a chlorophyll retention assay was performed to evaluate the salt tolerance in WT and *Ssb_Xoc_* transgenic plants when they were exposed to 0, 200, and 400 mM NaCl. The results showed that when exposed to 200 mM NaCl, the chlorophyll contents of WT, OE1 and OE9 were 54.3, 65.9, and 67%, respectively, and they were further reduced to 23.3, 39.1, 49% during treatment with 400 mM NaCl, respectively (**Figures 7B,C**). Thus, chlorophyll retention was higher in *Ssb_Xoc_* transgenic lines than in WT, suggesting that overexpression of *Ssb_Xoc_* improved salt tolerance in transgenic *N. benthamiana*.

### SSB_Xoc_ Decreases MDA Level and Increases Proline Content During Salt Stress

**FIGURE 8:**
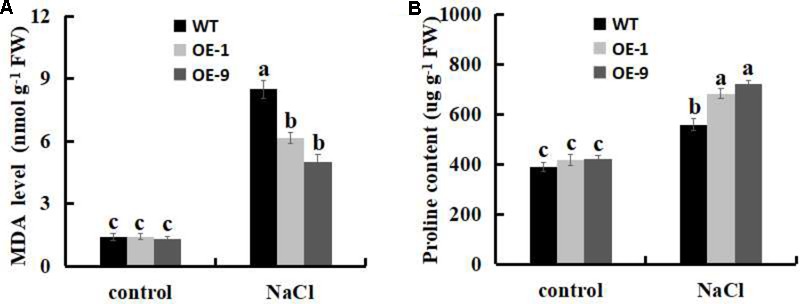
Analysis of physiological indicators of lipid peroxidation (MDA) and proline in WT and *Ssb_Xoc_* transgenic *N. benthamiana* plants under salt stress. **(A)** MDA levels and **(B)** Proline contents in WT and transgenic plants after treatment with 0 and 200 mM NaCl for 4 d. Error bars represent SD, and values with different letters are significant at *P* < 0.05.

Malondialdehyde level has been used as a biological marker for the end-point of lipid peroxidation (Yoshimura et al., 2004; Wang et al., 2017), thus, we measured the MDA levels in WT and *Ssb_Xoc_* transgenic plants under the salt stress. No differences were observed in MDA contents between WT and *Ssb_Xoc_* transgenic lines when exposed to 0 mM NaCl, however, MDA level was significantly higher in WT than in transgenic lines after treatment with 200 mM NaCl (**Figure 8A**), indicating that lipid peroxidation, and hence membrane damage, was lower in transgenic *N. benthamiana*.

The accumulation of proline in plant cells is indicative of enhanced salt stress tolerance (Vinocur and Altman, 2005; Miller et al., 2010; Wang et al., 2015). Therefore, we evaluated the proline contents of leaves in WT and transgenic lines when they were exposed to salt stress. As shown in **Figure 8B**, no obvious differences were observed in proline contents between WT and *Ssb_Xoc_* transgenic lines without NaCl treatment, however, in transgenic lines, proline contents significantly increased more than in WT after treatment with 200 mM NaCl (**Figure 8B**). Thus, the increased proline contents implies the improved salt tolerance in *Ssb_Xoc_* transgenic plants.

### SSB_Xoc_ Improves the Expression of Stress-Related Genes During Salt Stress

**FIGURE 9:**
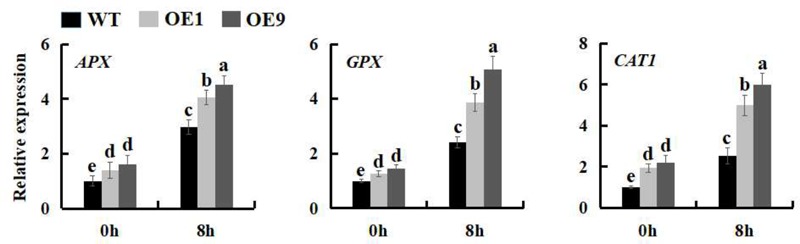
Expression levels of stress-related genes in WT and *Ssb_Xoc_* transgenic *N. benthamiana* plants under salt stress. Two-month-old seedlings were treated with 0 and 200 mM NaCl, and after 8 h, the leaves were sampled to extract the total RNA to synthesize cDNA. The expression levels of *APX, GPX* and *CAT1* genes were analyzed by qRT-PCR. Error bars represent SD, and values with different letters are significant at *P* < 0.05.

More and more results demonstrated that plants modulate the expression of many stress-related genes as an adaptation to environmental stress (Umezawa et al., 2006; Chinnusamy et al., 2007; Hirayama and Shinozaki, 2010; Bharti et al., 2016). To better understand the mechanistic basis of salt tolerance in *Ssb_Xoc_* transgenic lines, we measured the expression levels of three stress-related genes, *APX, GPX* and *CAT1*, which separately encode ascorbate peroxidase, glutathione peroxidase, and catalase. As shown in the **Figure 9**, *Ssb_Xoc_* transgenic plants displayed a higher basal expression level of the three genes as compared to WT without salt stress; under 200 mM NaCl treatment, the expression levels of these three genes were all significantly enhanced in WT and *Ssb_Xoc_* transgenic lines, while they were increased more in the latter (**Figure 9**). These results indicated that *Ssb_Xoc_* transgenic *N. benthamiana* plants improved salt tolerance through up-regulating the expression of stress-related genes.

## Discussion

### SSB_Xoc_ Improves Plant Growth in Transgenic *N. benthamiana*

We previously demonstrated that the exogenous application of SSB_Xoc_ enhanced growth of tobacco and *Arabidopsis* (Li et al., 2013). In this study, we cloned *Ssb_Xoc_* gene from *X. oryzae* pv. *oryzicola* and transferred it into *N. benthamiana* via *Agrobacterium*-mediated transformation. Two *Ssb_Xoc_* transgenic lines (OE1 and OE9) were characterized, and both of them showed improved root elongation and enhanced foliar growth as compared to WT plants (**Figures 2A–C**). Previous study reported that the expansin family genes were required for leaf growth (Goh et al., 2012) and EIN2 participated in the process of plant development and positively regulated the ethylene signaling pathway (Johnson and Ecker, 1998; Wang et al., 2002). Thus, we measured the expression levels of one expansin-encoding gene, *EXPA1* and *EIN2* to investigate whether or not they were changed in *Ssb_Xoc_* transgenic plants. As shown in **Figure 2D**, the transgenic lines exhibited higher expression of the two genes in comparison with WT, which further confirmed the growth phenotypes of *Ssb_Xoc_* transgenic plants (**Figure 2C**).

### SSB_Xoc_ Transgenic Plants Exhibit Potentiated Defense Responses

Many studies have demonstrated that the activation of MAPK-dependent signaling cascades (Nakagami et al., 2005), ROS, and defense gene expression (Nürnberger, 1999; Gómez-Gómez and Boller, 2000) occurs in most plant-pathogen interactions, which leads to an improved defense resistance. During this process, the activities of defense enzymes are usually triggered initially in the plant-pathogen interactions (Ramamoorthy et al., 2002), and the speed of these defense responses is faster in incompatible interactions (Kombrink and Somssich, 1995). Corresponded to these conclusions, the expression of *PR* genes was increased more in WT and *Ssb_Xoc_* transgenic *N. benthamiana* when the tested plants were inoculated with *Pst* DC3000 rather than *P. s. tabaci*, excepting for the expression of *SGT1* in WT plants.

Reactive oxygen species, e.g., H_2_O_2_ and O_2_^-^, are primarily produced at the site of attempted pathogen invasion in plant cells (Nanda et al., 2010; Jwa and Hwang, 2017) and are indicative of pathogen recognition and activation of plant defense responses (Lamb and Dixon, 1997; Torres, 2010). Up to now, more and more researches demonstrate that exogenous harpins, including Hpa1, induce ROS accumulation in tobacco and *Arabidopsis* cell cultures (Desikan et al., 1998; Andi et al., 2001; Samuel et al., 2005; Zou et al., 2006; Li et al., 2013; Choi et al., 2013). In the current study, the ROS level was higher in *Ssb_Xoc_* transgenic plants after inoculation with Hpa1 protein and the incompatible pathogen, *Pst* DC3000 (**Figure 3B**), which finally led to an earlier HR (**Figure 3C**). In addition, the expression of *PR* genes, HR marker genes, and *MPK3* gene was also higher in transgenic lines than that in WT after inoculation with *Pst* DC3000 for 6 h (**Figure 4**). In a word, the higher levels of ROS and the improved expression of defense-related genes in *Ssb_Xoc_* transgenic plants were consistent with the rapid elicitation of the HR. Previous studies have shown that the HR generally appears within 24 h after inoculation with an incompatible pathogen or harpin (Wei et al., 1992; He et al., 1993). In this study, we inoculated *N. benthamiana* plants with reduced levels of Hpa1 (10 μg ml^-1^) and *Pst* DC3000 (OD_600_ = 0.01). Using this approach, we discovered that *Ssb_Xoc_* transgenic plants were more sensitive to the two eliciting agents accompanied with the increased expression of *Ssb_Xoc_* gene in transgenic plants, finally leading to producing a stronger HR at 24 hpi than WT plants (**Figure 3C** and Supplementary Figure S1).

Previously, *Nicotiana tabacum* cv. Xanthi plants infiltrated with SSB_Xoc_ displayed an improved resistance to the tobacco pathogen, *Alternaria alternata* (Li et al., 2013). In the current study, another pathogenic bacterium, *P. s. tabaci*, was used to inoculate WT and *Ssb_Xoc_* transgenic *N. benthamiana* plants. The results showed that *Ssb_Xoc_* transgenic lines had the higher basal transcription levels of *PR1a, PR2, PR4*, and *SGT1* as compared to WT plants. After inoculation with *P. s. tabaci* for 6 h, expression of *PR* genes was significantly increased more in *Ssb_Xoc_* transgenic lines, and this was accompanied by a slight reduction in pathogen growth than WT plants (**Figures 5, 6**), suggesting the enhanced bacterial resistance in *Ssb_Xoc_* transgenic *N. benthamiana*.

### *Ssb_Xoc_* Transgenic Plants Show Improved Salt Tolerance

Salt stress has many deleterious effects on plant growth and development, and inhibits seed germination, chlorophyll retention, root length, and fructification (Zhang et al., 2006; Sui et al., 2017; Liang et al., 2018). We initially used percent seed germination and chlorophyll retention to evaluate salt tolerance and discovered that *Ssb_Xoc_* transgenic *N. benthamiana* plants displayed higher levels of germination rates and chlorophyll contents when exposed to different concentrations of NaCl (**Figure 7**), indicating the enhanced salt tolerance of transgenic plants.

We next used MDA and proline as bioindicators to investigate the salt stress tolerance of *Ssb_Xoc_* transgenic *N. benthamiana* in the present study. MDA is the main product of membrane lipid peroxidation when plants are under salt stress (Liang et al., 2018), and MDA content has been used as a biological marker for the degree of membrane damage (Yoshimura et al., 2004; Wang et al., 2017). In our current study, we noted lower MDA levels in *Ssb_Xoc_* transgenic lines than in WT under salt stress condition (**Figure 8A**), suggesting that the degree of lipid peroxidation was lower in transgenic lines. Proline is an important osmotic adjustment compound in plant cells and plays a crucial role in protecting macromolecules and cellular membranes (Singh et al., 2000; Miller et al., 2010; Liang et al., 2018). The elevated accumulation of proline in plant cells is indicative of enhanced salt stress tolerance (Vinocur and Altman, 2005; Miller et al., 2010). In our research, we also observed a significant increase of proline contents in transgenic lines as compared to WT plants (**Figure 8B**), implying the enhanced salt tolerance in *Ssb_Xoc_* transgenic *N. benthamiana*.

During salt stress, the concentration of ROS increases to a potentially toxic level. To overcome H_2_O_2_-related cellular damage, organisms produce various antioxidant enzymes, including ascorbate peroxidase (APX), glutathione peroxidase (GPX), and catalase (CAT) (Ozyigit et al., 2016). The improved expression of *APX, GPX*, and *CAT* was correlated with the increased salt tolerance in both WT and transgenic plants (Mishra and Tanna, 2017). In the current study, the expression of *APX, GPX* and *CAT1* was higher in *Ssb_Xoc_* transgenic lines than in WT both under normal and salt stress conditions, particularly in OE9 line, which had the higher expression level of *Ssb_Xoc_* gene (**Figure 9** and Supplementary Figure S1). Thus, in addition to the elevated proline levels, the activities of ROS-scavenging enzymes were also increased in transgenic lines, finally leading to the enhanced tolerance to salt stress in *Ssb_Xoc_* transgenic plants.

However, little is known about the mechanisms how harpins and SSB protein trigger many similar beneficial effects on plants, though both harpins (including Hpa1) and SSB protein have some common features as mentioned elsewhere in this report. We hypothesize that, SSB_Xoc_, like Hpa1, is translocated through the T3SS into plant cells, and possibly also perceived in plant apoplast, where it is recognized by unknown receptor(s) that recruit other proteins to activate downstream signal transduction cascades for HR induction, leading to expression of Eth-dependent genes for plant growth and SA- or JA-dependent genes for plant defense. Nevertheless, the discovery of harpin or SSB receptors in plants is the key to understand this point.

## Conclusion

Our previous research displays that SSB from *X. oryzae* pv. *oryzicola* shares many features in common with the harpin Hpa1. Similar to Hpa1, SSB_Xoc_ is an acidic glycine-rich, heat-stable protein that lacks cysteine residues, which can also stimulate an HR in tobacco (Li et al., 2013). Thus, in many aspects, SSB_Xoc_ functions in a similar manner to harpins. The present studies have shown that SSB proteins in *Escherichia coli* are found to bind to ssDNA in a sequence-independent manner, and protect ssDNA from forming secondary structures and subsequent degradation by nucleases (Shereda et al., 2008; Bianco, 2017). Although SSB_Xoc_ clearly functions as a harpin, it may also have additional functions that are similar to SSB in *E. coli.* Thus, it is tempting to speculate that SSB_Xoc_ may impart increased resistance to ROS in transgenic plants via the protective roles, such as the increased repair ability of single-stranded breaks due to oxidative stress. In a word, regardless of the precision mechanisms in the current study, SSB_Xoc_ has the potentials in improving plant growth, imparting enhanced disease resistance and improving salt tolerance in *N. benthamiana*.

## Author Contributions

YC and GC designed the experiments. YC performed most of the experiments and analyzed most of the data. MY detected the H_2_O_2_ contents and analyzed part of the data. WM provided some experimental methods. YS constructed the plasmid of pCAMBRIA2300-*Ssb_Xoc_*. GC and YC wrote the manuscript.

## Conflict of Interest Statement

The authors declare that the research was conducted in the absence of any commercial or financial relationships that could be construed as a potential conflict of interest.
