# Supplementary material for: Overexpression of SSBXoc, a Single-Stranded DNA-Binding Protein From Xanthomonas oryzae pv. oryzicola, Enhances Plant Growth and Disease and Salt Stress Tolerance in Transgenic Nicotiana benthamiana
Source: Front Plant Sci. 2018 Jul 5;9:953. doi: 10.3389/fpls.2018.00953 (PMC6041465; doi:10.3389/fpls.2018.00953)
Supplement: Supplementary file 1 [file Image_1.PDF]

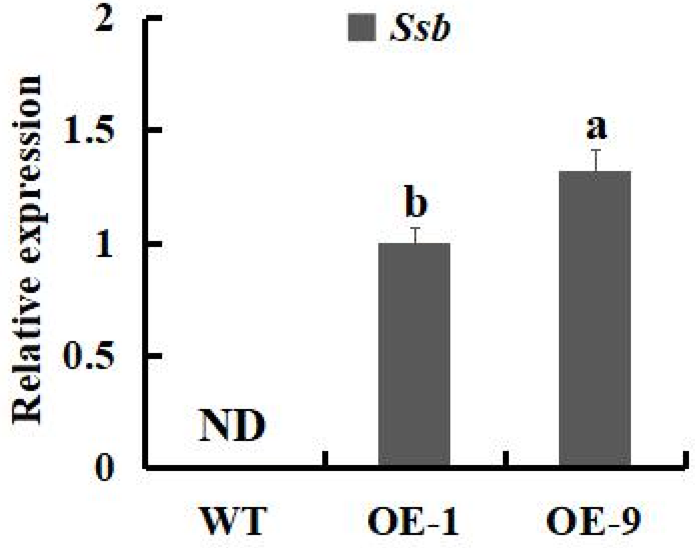

Supplementary Figure S1. Expression analysis of *Ssb*$_{Xoc}$ gene in *Ssb*$_{Xoc}$ transgenic *N. benthamiana*. The leaves of two-month-old WT and T1 *Ssb*$_{Xoc}$ transgenic *N. benthamiana* were sampled to extract the total RNA to synthesize cDNA, and the expression level of *Ssb*$_{Xoc}$ gene was analyzed by qRT-PCR. Error bars represent SD, and values with different letters are significant at $P < 0.05$.

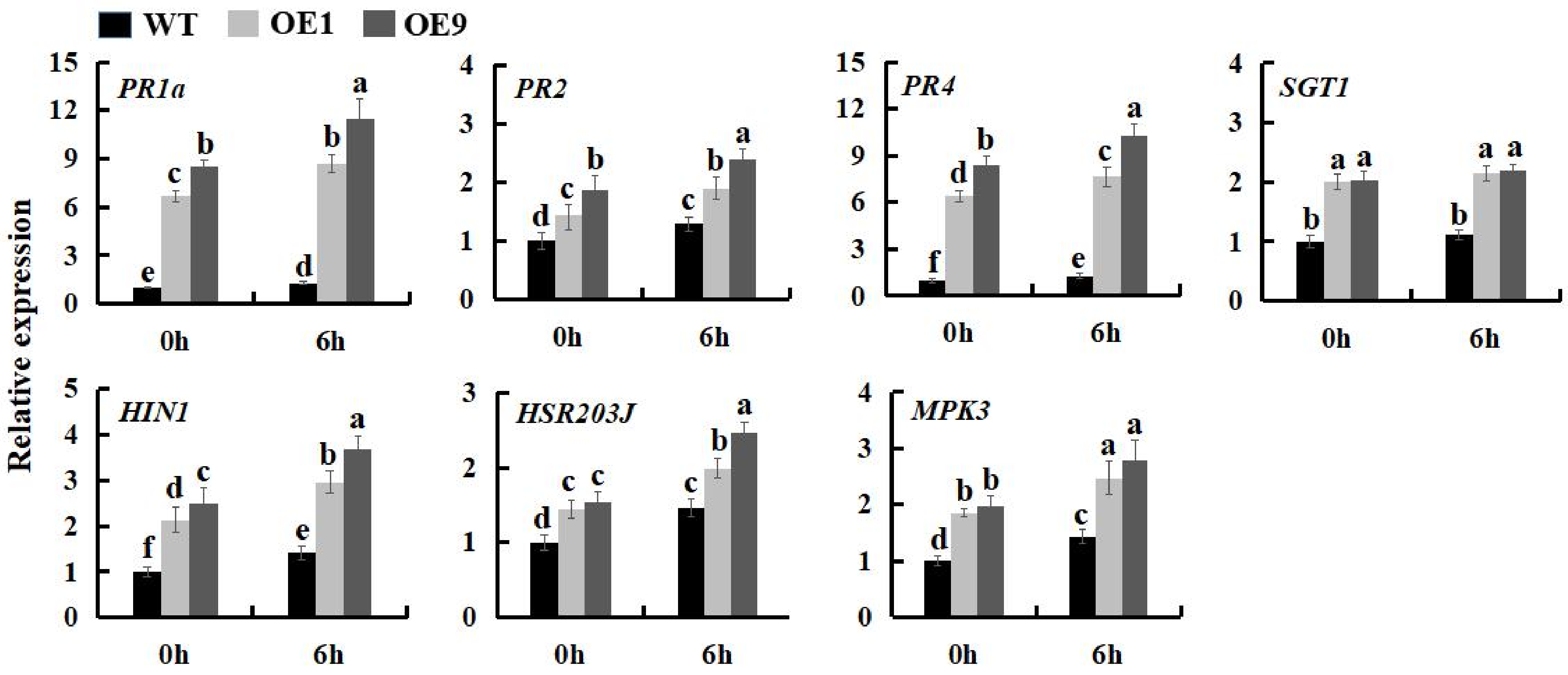

Supplementary Figure S2. Expression analysis of defense-related genes in WT and *Ssb$_{Xoc}$* transgenic *N. benthamiana* mock-inoculated with 10 mM MgCl$_2$. Two-month-old seedlings were inoculated with 10 mM MgCl$_2$. At 0 and 6 hpi, the leaves were sampled to extract the total RNA to synthesize cDNA, and the expression levels of *PR1a*, *PR2*, *PR4*, *SGT1*, *HIN1*, *HSR203J* and *MPK3* genes were analyzed by qRT-PCR. Error bars represent SD, and values with different letters are significant at $P < 0.05$.
